# Association of *IL1R1* Coding Variant With Plasma-Level Soluble ST2 and Risk of Aortic Dissection

**DOI:** 10.3389/fcvm.2021.710425

**Published:** 2021-08-02

**Authors:** Wenxi Jiang, Xue Wang, Pei Gao, Fengjuan Li, Ke Lu, Xin Tan, Shuai Zheng, Wang Pei, Meiyu An, Xi Li, Rong Hu, Yongliang Zhong, Junming Zhu, Jie Du, Yuan Wang

**Affiliations:** ^1^Key Laboratory of Remodeling-Related Cardiovascular Diseases, Ministry of Education, The Collaborative Innovation Center for Cardiovascular Disorders, Beijing Anzhen Hospital, Capital Medical University, Beijing, China; ^2^Department of Vascular Biology, Beijing Institute of Heart, Lung and Blood Vessel Disease, Beijing, China; ^3^Department of Epidemiology and Biostatistics, School of Public Health, Peking University, Beijing, China

**Keywords:** *IL1R1* gene, soluble ST2, aortic dissection, ST2-related genes, risk factor

## Abstract

**Objective:** Aortic dissection (AD) is characterized by an acute onset, rapid progress, and high mortality. Levels of soluble ST2 (sST2) on presentation are elevated in patients with acute AD, which can be used to discriminate AD patients from patients with chest pain. sST2 concentrations were found to be highly heritable in the general population. The aim of this study was to investigate the associations of variations in ST2-related gene expression with sST2 concentrations and AD risk.

**Methods:** This case-control study involving a total of 2,277 participants were conducted, including 435 AD patients and age- and sex-matched 435 controls in the discovery stage, and 464 patients and 943 controls in the validation stage. Eight ST2-related genes were selected by systematic review. Tag single-nucleotide polymorphisms (SNPs) were screened out from the Chinese population of the 1,000 Genomes Database. Twenty-one ST2-related SNPs were genotyped, and plasma sST2 concentrations were measured.

**Results:** In the discovery stage, rs13019803 located in *IL1R1* was significantly associated with AD after Bonferroni correction (*p* = 0.0009) and was correlated with circulating sST2 levels in patients with type A AD(AAD) [log-sST2 per C allele increased by 0.180 (95%) CI: 0.002 – 0.357] but not in type B. Combining the two stages together, rs13019803C was associated with plasma sST2 level in AAD patients [log-sST2 increased by 0.141 (95% CI: 0.055–0.227) for per C allele]. Odds ratio of rs13019803 on the risk of AAD is 1.67 (95% CI: 1.33–2.09).

**Conclusions:** The *IL1R1* SNP rs13019803C is associated with higher sST2 levels and increased risk of AAD.

## Introduction

Aortic dissection (AD) is an important cause of cardiovascular death characterized by acute onset and rapid progress with poor prognosis and high mortality (1). Acute AD is a fatal clinical emergency, with an untreated mortality rate of 1–2% per hour after symptom onset and almost 50% in the first week (2). Therefore, identifying those members of the population with higher risk to AD is very important. Contributing factors of AD are diverse, including male sex, advanced age, smoking, hypertension, and genetic factors (3). Although the pathogenesis of AD remains unclear, genetic variation plays a critical role, and the American College of Cardiology Foundation and the American Heart Association recommended that identification of the genetic variations leading to these aortic diseases has the potential for early identification of individuals at risk (4).

AD is a multifactorial disease whose primary pathology is connective tissue degeneration of the medial layer of the aorta. During the early phase of aortic damage, a large number of vascular injury-related proteins are produced in response to injury of vascular smooth muscle and elastic laminae (5–7). Soluble (s)ST2 is also regulated by vascular injury and is rapidly secreted into the circulation after stress and pro-inflammatory stimulation. We have used it to discriminate acute AD from other diseases presenting with acute chest pain (8). In the early stages of AD, sST2 elevated levels reflect the degree of vascular injury (9, 10). sST2 also participates in adverse remodeling of blood vessels by stimulating expression of type I collagen, fibronectin, and profibrotic factors (11).

In previous studies, it was found that the concentration of sST2 varies greatly in AD patients (8). The level of soluble ST2 was affected by age, gender, clinical manifestations, and genetic variation in the general population (12). Despite being correlated with multiple traditional cardiovascular risk factors, soluble ST2 concentrations were found to be highly heritable in the Framingham Heart Study population, in which clinical factors accounted for only 14% of the interindividual variation in soluble ST2 concentrations, and genetic factors accounted for up to 45% of the remaining variation. Therefore, although sST2 is affected by different clinical phenotypes, genetic factors may also play an important role in determining the circulating level of patients.

Variation in genes encoding vascular injury-associated proteins is known to associate increased risk for AD, such as matrix metalloproteinases(MMPs), SMAD4, and interleukin 6 (IL-6) genes (13–15). However, it is unclear whether variation in ST2 gene expression is related to AD risk. Determination of genetic variants that significantly affect sST2 concentrations and AD risk would be helpful in the early identification of high-risk populations to achieve better clinical outcomes.

The aim of this study, therefore, was to investigate whether sST2-related gene polymorphisms are associated with increased sST2 concentrations and higher risk of AD.

## Materials and Methods

### Participants

**Figure 1:**
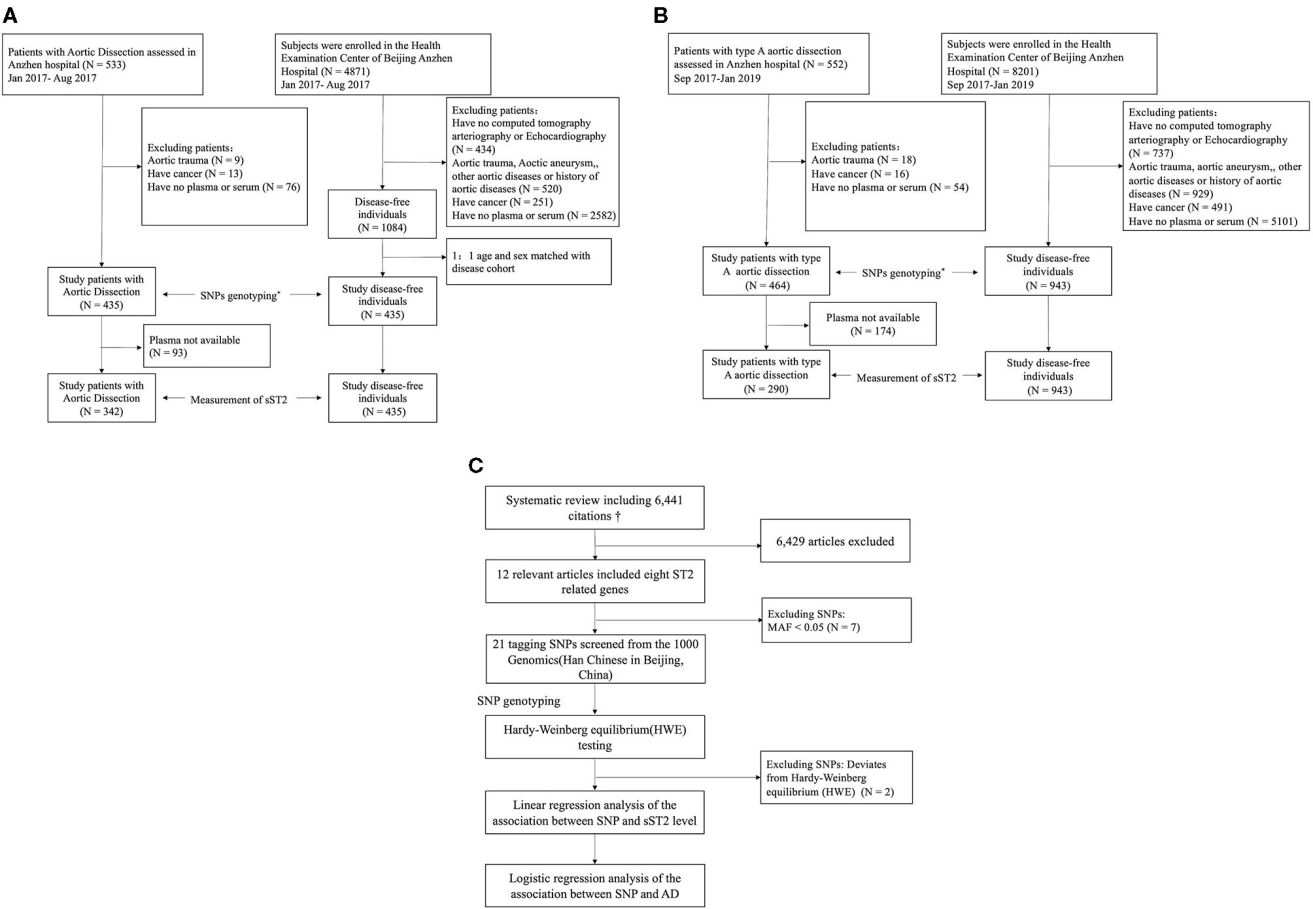
Overall study design. **(A)** Work flow of the selection subjects for the discovery stage. **(B)** Work flow of the selection subjects for the validation stage. **(C)** Selection of tag SNPs. AD, aortic dissection; SNP, single-nucleotide polymorphism. *The SNP screening design is shown in **(C)**. ^†^See Supplementary Materials for system review details.

The overall study design is shown in Figure 1. This study included AD patients and controls who had visited Anzhen Hospital, Beijing, China, between January 2017 and January 2019. The study comprised a discovery stage including participants from January 2017 to August 2017 and a validation stage including participants from September 2017 to January 2019. All patients who were referred to the surgical service for the evaluation and management of AD were included. All patients with AD had image information from echocardiograms and computed tomography to confirm the final diagnosis. The diagnosis of AD was based on the detection of intimal flaps in the aorta (16). The Stanford classification was applied to determine the types of AD. With this classification system, type A AD is defined as an intimal tear involving the ascending aorta, whereas type B AD is not (17). Patients were excluded if they met any of the following criteria: (a) received packed red blood cells, whole blood, or platelets fewer than 10 days before the blood sample was taken; (b) aortic trauma; (c)with active cancer; and (d) entered the hospital for checkups after surgery or undergone open or endovascular treatment in temporal proximity fewer than 1 year. A patient's aorta was considered aneurysmal when its diameter was >1.5 times the normal diameter, based on the patient's sex, age, and body size. The acute phase, subacute phase, and chronic phase are defined as occurring within 2 weeks, 2 weeks to 2 months, and more than 2 months after the onset of initial symptoms, respectively. We defined the thoracic aorta as the portion of the vessel located above the diaphragm, including the ascending aorta, the aortic arch, and the descending thoracic aorta, and we defined the abdominal aorta as the portion of the vessel located below the diaphragm, including the suprarenal and infrarenal segments. Malperfusion syndromes were subclassified as (1) coronary: ischemic electrocardiographic changes, elevation of troponin levels, and regional wall motion abnormalities on echocardiography; (2) hepatic: increased liver enzymes; (3) mesenteric: abdominal tenderness, bowel paralysis, lactate acidosis; (4) renal: decreased urine output and increased creatinine; (5) nervous system: transient ischemic attack, stroke, paraplegia/paraparesis, limb (loss of pulses, clinical signs of limb ischemia). The degree of aortic injury was defined based on previous studies, including *in situ* aortic and distal aorta injury. Severely involved aortic root type: (a) the diameter of the aortic sinus was between 35 and 50 mm with severe disruption of the sinotubular junction, (b) the diameter of the aortic sinus was 50 mm or greater, and (c) severe aortic insufficiency. Severely involved in the distal aorta are the following (dependent upon the extent and extension of aortic dissection extending beyond the ascending aorta and arch): (a) the primary tear located in the transverse arch or the descending aorta; (b) aneurysm formation in the aortic arch or the distal aorta (>40 mm); (c) involvement, aneurysm formation, and occlusion of the brachiocephalic artery; (d) Marfan syndrome; (e) or sleeve-like striping. Mild involved type is when the primary tear was located in the ascending aorta without the above characteristics (18).

Controls were participants without aortic diseases who underwent routine annual health checks and who had a blood sample saved at the hospital. Controls without aortic dissection were selected from potentially eligible subjects who had chest or abdominal images admitted during the same period. They were excluded from the study if they met any of the following criteria: (a) received packed red blood cells, whole blood, or platelets fewer than 10 days before the blood sample was taken; (b) with aortic trauma, aortic aneurysm, other aortic disease or family history of aortic disease; and (c) active cancer. In the discovery stage, a subset of healthy controls was then randomly selected from the database, matched 1:1 with the selected cases by age (3 years), sex, and geographic region.

None of the subjects were related to each other, and all provided written informed consent for study participation. Baseline characteristics of the subjects were collected from medical records and confirmed by the study physicians. The height and weight of the patients were measured directly or self-reported, and body mass index (BMI) was calculated. The study included eligible patients from the DPANDA aortic aneurysm and/or dissection registry study at Anzhen Hospital (NCT03233087 in clinicaltrials.gov). This study was approved by the Beijing Anzhen Hospital Ethics Review Board.

### Tag Single-Nucleotide Polymorphisms Selection and Genotyping

All reported genes related to sST2 concentrations were identified from a systematic review (see details in Supplementary Text 1; Supplementary Text 2; Supplementary Table 1; Supplementary Figure 1). Polymorphisms around the eight candidate loci (*IL1RL1, IL1RL2, IL1R1, IL18R1, IL18RAP, SLC9A2, SLC9A4*, and *SH3YL1*) drawn from the 1000 Genomes Project (Han Chinese in Beijing, China, https://www.internationalgenome.org) were analyzed with Haploview (v4.1, Broad Institute, Boston, MA, USA). Markers were selected from the genomic region of each locus, including 5 kb up- and downstream of the coding region. Following filtering of the marker set to exclude rare alleles (minor allele frequency <0.05), the tagger algorithm was used to select markers from the 1,000 Genomes Project database. This identified a set of SNPs that efficiently captured genetic variation at each locus so that all untyped variants had a high correlation (*R*^2^ > 0.8) with one member of the typed set. If there is a linkage balance between SNPs in the same gene in the screening process, the reported SNPs will be preferred. Genotyping results were tested for Hardy–Weinberg equilibrium. Nineteen of 21 SNPs did not deviate significantly from the Hardy–Weinberg equilibrium (*p* > 0.001), so they were included in the subsequent analysis (Supplementary Table 2).

DNA from whole blood was isolated using a commercial DNA isolation kit (BioTeKe Corporation, Beijing, China). Genotyping was performed by mass spectrometry (Massarray System 4; Agena Inc., San Diego, CA, USA). Polymerase chain reaction (PCR) and detection primers were designed using the MassARRAY Assay Design software (Sequenom). The DNA samples were amplified by multiplex PCR reactions. The terminator nucleotides were desalted and added into a 384-element SpectroCHIP array. Then, allele detection was performed using a time-of-flight (MALDI-TOF) SpectroReader mass spectrometer (Sequenom). Last, the mass spectrograms were analyzed for peak identification using the Typer Analyzer software (Sequenom), and for quality control, the missing genotype rate of each SNP was set to lower than 10%.

### sST2 Measurements

Plasma samples were collected from subjects at admission and stored at −80°C. Circulating sST2 was measured using a DuoSet ELISA kit (DY523B-05; R&D Systems, Minneapolis, MN, USA) according to the instructions of the manufacturer (19–21). The limit of detection for sST2 was 0.019 ng/ml, with a mean intra-assay coefficient of variation of <6.0% and mean inter-assay coefficient of variation of <9.5%. Detailed procedures as well as the comparison between assay methods for sST2 are described in Supplementary Text 3.

### Statistical Analyses

Continuous variables in the patients' information were expressed as mean [standard deviation (SD)] or median (interquartile range) for skewed variables (e.g., sST2). The two-sample *t*-test was used to compare continuous variables (log-transformed where appropriate). Categorical variables were presented as counts and percentages, and differences were assessed using the χ^2^ test or Fisher's exact test. Soluble ST2 concentrations were log-transformed because of their right-skewed distribution. Associations of genetic variants and sST2 concentrations were tested using linear regression under an additive genetic model. Bonferroni correction was used for multiple comparison adjustments of the *p*-values. Analyses were performed using Stata version 15.1 (Stata Corp, College Station, TX, USA) and Haploview version 4.2 (Daly Lab, Cambridge, MA, USA) software.

## Results

### Study Populations

This study included 899 AD patients and 1,378 controls. The discovery stage included 435 AD patients and 435 controls from Anzhen Hospital in China between January 2017 and August 2017, whereas the validation study included 464 type A AD patients and 943 controls recruited between September 2017 and January 2019 (Figure 1; Supplementary Text 4; Supplementary Figures 2, 3). sST2 concentrations were measured in 632 AD patients with plasma available. Baseline characteristics of all participants are shown in Table 1. Compared with controls, AD patients had a significantly higher body mass index (BMI; 25.56 ± 3.67 vs. 24.52 ± 3.16 in the discovery stage, and 25.99 ± 3.62 vs. 23.68 ± 3.27 in the validation stage; both *p* < 0.05), significantly more were smokers (42.8 vs. 27.1% in the discovery stage and 44.0 vs. 19.5% in the validation stage; both *p* < 0.05), and had significantly more frequent hypertension (68.7 vs. 21.4% in the discovery stage and 71.6 vs. 18.6% in the validation stage; both *p* < 0.05). sST2 levels were elevated at 34.63 ng/ml (median, IQR: 15.85–74.90) in the patients with AD compared with 8.09 ng/ml in controls (median, IQR: 6.18–10.70) in the discovery stage, and 53.64 ng/ml (median, IQR: 25.03–120.08) in the patients with type A AD compared with 7.98 ng/ml (median, IQR: 5.87–10.16) in controls in the validation stage. No other differences were observed between the two groups. Among the 899 AD patients, there were 652(72.5%) patients with type A aortic dissection and 247 (27.5%) patients with type B.

**Table 1:** Summary of participant characteristics.

	**Discovery stage**		**Validation stage**	
	**Controls (*n* = 435)**	**AD patients (*n* = 435)**	**Controls (*n* = 943)**	**Type A AD patients (*n* = 464)**
Age	48.88 (11.91)	48.95 (12.01)	42.56 (12.11)	50.03 (11.78)*
Sex (male)	330 (75.9%)	330 (75.9%)	474 (50.3%)	361 (77.8%)*
Smoke (current)	118 (27.1%)	186 (42.8%)*	184 (19.5%)	204 (44.0%)*
Diabetes (yes)	18 (4.1%)	24 (5.5%)	26 (2.8%)	21 (4.5%)
Hypertension (yes)	93 (21.4%)	299 (68.7%)*	175 (18.6%)	332 (71.6%)*
BMI (kg/m^2^)	24.52 (3.16)	25.56 (3.67)*	23.68 (3.27)	25.99 (3.62)*
CAD (yes)	26 (6.0%)	34 (7.8%)	33 (3.5%)	38 (8.2%)*
Hyperlipidemia (yes)	88 (20.2%)	77 (17.7%)	204 (21.6%)	90 (19.4%)
sST2 (ng/ml)	8.09 (6.18,10.70)	34.63 (15.85,74.90)*^a^	7.98 (5.87,10.16)	53.64 (25.03, 120.08)*^b^
Log-sST2	0.90 (0.18)	1.55 (0.49)*^a^	0.88 (0.18)	1.73 (0.48)*^b^

* *p < 0.05*.

a *Only 342 AD patients in the discovery stage had sST2 concentrations measured with plasma available*.

b *Only 290 type A AD patients in the validation stage had sST2 concentrations measured with plasma available*.

*AD, aortic dissection; BMI, body mass index; CAD, coronary artery disease*.

### Selection of Single-Nucleotide Polymorphisms With Soluble ST2 Concentrations in Control and Aortic Dissection

The association of these 19 SNPs with sST2 concentrations was reported in the discovery stage of the study. Of these SNPs, 15 located in seven genes (rs1921622, rs3821204, rs6751967, and rs12712135 in *IL1RL1*; rs887971 and rs1558650 in *IL18RAP*; rs4241211, rs4851608, rs1468788, and rs11692304 in *SLC9A4*; rs3771172 and rs2241116 in *IL18R1*; rs13019803 in *IL1R1*; rs17775170 in *SLC9A2*; and rs2241132 in *IL1RL2*) were shown to be significantly associated with sST2 concentration in controls (*p* < 0.0026; Supplementary Table 3).

The genotype distribution of the 19 SNPs and chi-square analysis in the discovery stage is shown in Table 2. In controls, the C allele of rs13019803 (located in *IL1R1*) accounted for 82.4%, and the T allele accounted for 17.6%. In patients with AD, the C allele accounted for 88.0% and the T allele for 12.0%. The T allele of rs13019803, referring to the reverse strand, represents the alternative (minor) allele for this SNP. rs13019803 (located in *IL1R1*) was significantly associated with AD (*p* = 9.30E−04), even after a conservative Bonferroni adjustment (*p* < 0.0026).

**Table 2:** Genotype distribution and chi-square analysis in the discovery stage.

**SNP**	**Gene**	**Major allele/minor allele**	**Controls (*N* = 435)^a^**	**AD patients (*N* = 435)^a^**	*p*-value^*^
rs13019803	IL1R1	C/T	0.176	0.120	9.30E−04
rs2241132	IL1RL2	C/A	0.300	0.272	0.200
rs4988958	IL1RL1	T/C	0.131	0.154	0.175
rs11692304	SLC9A4	G/A	0.181	0.203	0.262
rs6751967	IL1RL1	T/C	0.133	0.151	0.283
rs2241116	IL18R1	C/A	0.179	0.174	0.784
rs3917296	IL1R1	A/G	0.152	0.174	0.209
rs3917254	IL1R1	G/A	0.236	0.201	0.077
rs1468788	SLC9A4	C/T	0.223	0.202	0.279
rs887971	IL18RAP	T/C	0.321	0.293	0.222
rs4851608	SLC9A4	C/T	0.320	0.315	0.836
rs4241211	SLC9A4	T/G	0.439	0.445	0.805
rs10167431	IL1RL2	C/T	0.338	0.352	0.565
rs1921622	IL1RL1	G/A	0.366	0.354	0.597
rs17775170	SLC9A2	G/A	0.181	0.174	0.690
rs3771172	IL18R1	C/T	0.306	0.264	0.054
rs3821204	IL1RL1	C/G	0.312	0.275	0.086
rs1558650	IL18RAP	T/A	0.459	0.449	0.703
rs12712135	IL1RL1	A/G	0.456	0.485	0.234

* *Significant after Bonferroni correction for 19 tests (p <0.0026)*.

a *Minor allele frequencies*.

*AD, aortic dissection*.

### Association of rs13019803 With Soluble ST2 Concentration in Aortic Dissection Patients

In the discovery stage, sST2 concentrations were measured in 173 type A AD patients and 169 type B AD patients. For all AD patients, rs13019803C is related to the sST2 level [beta(95% CI) = 0.106 (−0.006–0.219), *p* = 0.064]. Compared with type A AD, type B AD does not involve the ascending aorta and is less dangerous (22). We separately analyzed type A and type B patients. rs13019803 is only related to the level of sST2 in patients with type A [beta(95% CI) = 0.180(0.002–0.357), *p* = 0.048], but not in type B [beta(95% CI) = −0.033 (−0.160–0.093), *p* = 0.604]. The correlation between rs13019803 and sST2 concentration in AAD patients remained significant after adjusting for confounders [beta(95% CI) = 0.229(0.066–0.393), *p* = 0.006] (Table 3). Therefore, only patients with type A AD were included in the validation cohort.

**Table 3:** rs13019803 associated with sST2 concentrations in AD patients in the discovery stage.

	**Beta (95% CI)^a^**	***p*-value^a^**
**Discovery (** ***N*** **=** **342)**		
Model 1	0.106 (−0.006,0.219)	0.064
Model 2	0.126 (0.016,0.236)	0.025
Model 3	0.132 (0.031,0.234)	0.010
**Type A (** ***N*** **=** **173)**		
Model 1	0.180 (0.002,0.357)	0.048
Model 2	0.189 (0.012,0.366)	0.036
Model 3	0.229 (0.066,0.393)	0.006
**Type B (** ***N*** **=** **169)**		
Model 1	−0.033 (−0.160, 0.093)	0.604
Model 2	−0.031 (−0.151, 0.089)	0.607
Model 3	−0.020 (−0.130,0.091)	0.725

a *Linear regression for log-sST2*.

*Model 1: Unadjusted*.

*Model 2: Adjusted age, sex, BMI, hypertension, hyperlipidemia, diabetes, smoking, and cardiovascular disease*.

*Model 3: Adjusted age, sex, BMI, hypertension, hyperlipidemia, diabetes, smoking, cardiovascular disease, and acuity*.

Overall, a total of 463 among 652 type A AD (AAD) patients were measured for sST2 concentrations with plasma available. Patients were divided into two groups according to the median level of sST2. Patients with acute onset, severe chest and back pain, malperfusion, and severe distal aorta injury have higher sST2 levels (*p* < 0.05; Supplementary Table 4). Then, we added the analysis after adjusting the above variables to the discovery stage. In the discovery stage, the correlation between rs13019803 and sST2 concentration in AAD patients remained significant after adjusting for confounders (age, sex, BMI, hypertension, hyperlipidemia, diabetes, smoking, cardiovascular disease, acute, abrupt onset pain, severely involved distal aorta, and pre-operative presence of malperfusion) [beta(95% CI) = 0.198(0.042–0.353), *p* = 0.013] (Supplementary Table 5). In the validation stage, for each additional copy of the C-allele, log-sST2 was increased by 0.139 (95% CI: 0.022–0.257) in AAD patients. Overall, for each additional copy of the C-allele, log-sST2 was increased by 0.151 (95% CI: 0.054–0.248) in AAD patients. After adjusting for the confounding factors in AAD patients, the correlation between rs13019803 and sST2 concentration in AAD patients remained significant [0.141 (95% CI: 0.055–0.227), *p* = 0.001] (Table 4).

**Table 4:** Association rs13019803C between sST2 in type A AD patients (*N* = 463).

	**Model**	**Beta (95% CI)^a^**	***p*-value^a^**
Validation	Model 1	0.139 (0.022, 0.257)	0.020
	Model 2	0.133 (0.014, 0.252)	0.029
	Model 3	0.113 (0.008, 0.217)	0.035
Combined	Model 1	0.151 (0.054, 0.248)	0.002
	Model 2	0.155 (0.057, 0.252)	0.002
	Model 3	0.141 (0.055, 0.227)	0.001

a *Linear regression for log-sST2*.

*Model 1: Unadjusted*.

*Model 2: Adjusted age, sex, BMI, hypertension, hyperlipidemia, diabetes, smoking, and cardiovascular disease*.

*Model 3: Adjusted age, sex, BMI, hypertension, hyperlipidemia, diabetes, smoking, cardiovascular disease, acute, abrupt onset pain, severely involved distal aorta, and pre-operative presence of malperfusion*.

### Association of rs13019803 With Type A Aortic Dissection Risk

We investigated the association of the SNPs rs13019803 and risk of AAD according to three regression models (Table 5). In the validation stage, for each additional C allele of rs13019803, the odds ratio (OR) of AAD was 1.35 (95% CI: 1.08–1.68; *p* = 0.007). Furthermore, this association remained significant after adjusting for age, sex, BMI, coronary artery disease (CAD), hypertension, diabetes, and hyperlipidemia (OR = 1.42; 95% CI: 1.09–1.85; *p* = 0.010). Overall, the OR of AAD was 1.54 (95% CI: 1.27–1.86; *p* = 1.1E−5) for each additional C allele of rs13019803. This association remained significant after adjusting for confounding factors (OR = 1.67; 95% CI: 1.33–2.09; *p* = 1.0E−5). The genotype of rs13019803 distribution was not associated with confounding factors of AAD patients (*p* > 0.05), but with sST2 level (*p* < 0.05) (Supplementary Table 6). In addition, we performed a subgroup analysis with or without hypertension. The results showed that rs13019803 was associated with the risk of AD in patients with and without hypertension [OR (95% CI) = 1.71 (1.26–2.33), *p* = 0.001 with hypertension; OR (95% CI) = 1.59 (1.13–2.23), *p* = 0.007 without hypertension] (Supplementary Table 7).

**Table 5:** Odds ratio of rs13019803 on the risk of type A AD.

	**Model**	OR (95% CI)^*^	***p*-value**
Discovery^a^	Model 1	2.37 (1.57, 3.59)	3.9E−5
	Model 2	2.37 (1.57, 3.58)	4.2E−5
	Model 3	2.97 (1.82, 4.84)	1.2E−5
Validation	Model 1	1.35 (1.08,1.68)	0.007
	Model 2	1.34 (1.06,1.69)	0.015
	Model 3	1.42 (1.09,1.85)	0.010
Combined	Model 1	1.54 (1.27,1.86)	1.1E−5
	Model 2	1.52 (1.25,1.86)	2.8E−5
	Model 3	1.67 (1.33,2.09)	1.0E−5

* *C allele is risk allele*.

a *Only type A AD patients included*.

*Model 1: Unadjusted*.

*Model 2: Adjusted age, sex*.

*Model 3: Adjusted age, sex, BMI, hypertension, hyperlipidemia, diabetes, smoking, and cardiovascular disease*.

*OR, odds ratio; CI, confidence interval*.

## Discussion

In this study, we analyzed the relationship between ST2-related genes and circulating sST2 concentrations or AD. The C allele of *IL1R1* rs13019803 was associated with increased circulating sST2 concentrations and risk of AAD.

Genetic variation was verified to affect sST2 levels. In five independent GWAS studies, it was found that multiple SNPs in a 1 M area near *IL1RL1* were related to ST2 concentration (*p* < 5 × 10^−8^) (23–27). Our results in controls are consistent with those of other studies. In molecular studies, *IL1RL1* missense mutation-induced ST2 expression directly affects the ST2L signal and regulates ST2 promoter activity. Variants of *IL1RL1* can also increase the expression of sST2 by inducing the expression of IL-33 and IL1-β, and enhancing the reactivity of IL-33 (23). In this study, we found that the C allele of rs13019803 is not only associated with sST2 concentration in healthy people but also with high sST2 concentration in patients with aortic dissection. The MAF of rs13019803 is 0.162 in Anzhen hospital population, which is nearly the same with that of the CHB population [*T* = 29 (14.1%) vs. 740 (16.2%), *p* = 0.407] (Han Chinese in Beijing, China, https://www.internationalgenome.org).

We found that rs13019803 was associated with sST2 level in patients, independent of patients characters. Before the onset of AD, ST2-related gene variations may contribute to disease development by increasing ST2 production at transcriptional and translational levels (28, 29). The long-term accumulation of ST2 from genetic variations may induce inflammatory reactions of the aorta and systemic blood vessels, leading to severe vascular injury in patients with high ST2 level (10, 30–32). Soluble ST2 concentration may be affected by the expression of ST2-related genes, which is driven by genetic variation under stress conditions in AD patients and finally leads to the difference of sST2 level in patients (33). There is a significantly positive correlation between rs13019803 and serum sST2 in type A AD; however, it showed no correlation in type B AD. Compared with type A AD, type B AD does not involve the ascending aorta and has less hemodynamic stress (22). This indicates that there may be differences for underlining pathophysiology between type A and type B. The level of sST2 could be differently regulated by hemodynamics between type A and type B. ST2-related genes are attractive candidates for AD risk. Soluble ST2 acts as a decoy receptor for IL-33 and inhibits IL-33/ST2L signaling, thus, blocking its protective effect. sST2 may be involved in the occurrence and expansion of AD through injury and inflammation of smooth muscle cells (34). Variants in these genes are associated with a range of diseases risk (23, 35) and are involved in a variety of progressive diseases including CAD, hypertension, and asthma (28, 36). The long-term accumulation of ST2 from genetic variations may induce inflammatory reactions of the aorta and systemic blood vessels, leading to blood vessel damage in the smooth muscle cells of individuals without AD (10).

This study investigated 21 SNPs in seven ST2-related genes, but only rs13019803 in *IL1R1* was found to be associated with AAD. Rs13019803 is located in the intron region. In the Framingham study, rs13019803 is the top 10 SNP in the GWAS study with the concentration of sST2 and is the most strongly associated with sST2 in *IL1R1* gene (23). In GTEx studies, it is located in the 3′-promoter flaking region of the gene, which may regulate the expression of this gene region. Rs13019803 is not only related to the concentration of sST2 but also to the regulation of *IL1R1* and *IL18* gene expression (37, 38). This SNP was not found to be related to other phenotypes other than sST2 (39, 40). *IL1R1* encodes cytokine receptors belonging to the IL-1 receptor family, including IL-1α, IL-1β, and IL-33 (41). IL-1β is a multifunctional proinflammatory cytokine that binds to the IL1-R receptor on target cells (42). Recent studies reported that sST2 and IL-1β expression is strongly correlated and that both systems are associated with inflammation and vascular smooth muscle cell injury (43–45). Local IL-1β expression may be involved in AD development by upregulating matrix metallopeptidase (MMP)-2 and MMP-9 and increasing elastin fiber breaks (46, 47). IL-18 encoded by *IL18* gene may participate in AD by regulating macrophage differentiation and inducing SMC apoptosis induced by macrophages (48). Therefore, the genetic variation of rs13019803 may affect the whole IL1 axis, not only the role of sST2. Although rs13019803C showed an increased risk of AD accompanied by high levels of sST2 in the AD group, further studies are needed to determine whether IL1β or other factors are involved in the effect of IL1R1 on AD.

Accumulating evidence supports the conclusion that sST2 is a biomarker of vascular health with diagnostic and/or prognostic value in various cardiovascular diseases, including coronary artery disease, myocardial infarction, atherosclerosis, giant-cell arteritis, acute aortic dissection, and ischemic stroke (10). Genetic variations may affect the development and prognosis of cardiovascular diseases by regulating sST2 production. ST2-related genes may have the value of being added to the polygenic detection panel of AD risk. However, these *IL1R1* variants should still be interpreted with caution because recent studies suggest that a small number of potentially causal genetic findings can be selected for return into clinical practice (49). Nonetheless, molecular studies can help clarify the causative role of identified variants (50). Our findings suggest that genetic variations of biomarkers for acute elevation might also have potential value in disease. This finding also provides a possible explanation for patients with similar disease severity, but the level of circulating sST2 is of variety.

This study demonstrates that genetic polymorphisms affect plasma sST2 levels and AD risk. The findings of this study will help to explain the difference in circulating sST2 levels after onset of disease and early identification of high-risk patients. However, this study also has potential limitations. First, it was a single-center study with a relatively small sample size at the discovery stage, so may lack the ability to detect SNPs with small effects and/or low MAF. Second, the mechanisms by which genes affect sST2 concentrations are still not fully understood. Therefore, there is a clear need for further studies to better understand the underlying molecular mechanisms.

In conclusion, *IL1R1* variation is related to sST2 levels and AAD risk. This SNP is located in an intron and, therefore, is unlikely to be functional itself, but it may be in LD with a yet to be identified functional locus. This study supports the notion that ST2-related genes, in particular, the *IL1R1* gene, might play a role in AD etiology and development. Further research is needed to confirm whether it can be used for patient screening and stratification.

## Data Availability Statement

The original contributions presented in the study are publicly available. This data can be found here: ClinVar, SCV001652890–SCV001652908.

## Ethics Statement

This study was approved by the Beijing Anzhen Hospital Ethics Review Board. The patients/participants provided their written informed consent to participate in this study.

## Author Contributions

YW and JD conceived and designed the research. WJ and XW acquired the data. WJ and PG performed the statistical analysis. WJ drafted the manuscript. JD made critical revision of the manuscript for key intellectual content. FL, XT, and SZ collected and subpacked the plasma samples. XL, RH, YZ, and JZ evaluated the participants. KL, WP, and MA consummated the clinical information. All authors contributed to the article and approved the submitted version.

## Conflict of Interest

The authors declare that the research was conducted in the absence of any commercial or financial relationships that could be construed as a potential conflict of interest.

## Publisher's Note

All claims expressed in this article are solely those of the authors and do not necessarily represent those of their affiliated organizations, or those of the publisher, the editors and the reviewers. Any product that may be evaluated in this article, or claim that may be made by its manufacturer, is not guaranteed or endorsed by the publisher.

## Abbreviations

AD: aortic dissection
sST2: soluble ST2
SNP: single-nucleotide polymorphism
MAF: minor allele frequency
CI: confidence interval
IQR: interquartile range
CAD: coronary artery heart disease
HBP: high blood pressure
BMI: body mass index.
