# Supplementary material for: Association of IL1R1 Coding Variant With Plasma-Level Soluble ST2 and Risk of Aortic Dissection
Source: Front Cardiovasc Med. 2021 Aug 2;8:710425. doi: 10.3389/fcvm.2021.710425 (PMC8365023; doi:10.3389/fcvm.2021.710425)
Supplement: Supplementary file 1 [file Data_Sheet_1.docx]

**SUPPLEMENTAL MATERIAL**

**Supplemental Text S1.**

**Search strategy**

Publication database: MEDLINE

#1 ST2 OR sST2 OR IL33 OR IL1RL1 OR Suppression of tumorigenicity 2 OR Interleukin-33 OR DER4 OR FIT-1 OR IL33R OR ST2L OR ST2V OR T1 OR IL 33

#2 genetic OR variants OR Genome OR SNP OR GWAS OR polymorphism OR allele OR gene OR heritability

#3 "Humans"[Mesh]

Search: #1 AND #2 AND #3

Restrictions: None

Result: 6,441

**Supplemental Text S2. ST2-related SNPs selecting**

**Systematic review and screening of sST2 related genes.** According to the pre-specified search strategy, 6441 articles were retrieved(Supplementary Text S1). According to the inclusion exclusion criteria, 12 studies covering ST2 related genes or SNPs were finally included (Supplementary Table S1). The specific retrieval process is shown in Supplementary Figure S2. In these 12 studies, if one or more SNPs were reported to be related to ST2, the genes in which these SNPs are located were included as ST2-related genes (P < 5×10^-8^ was used as the threshold in GWAS study, and other studies are based on the threshold used in the studies). A total of eight ST2-related genes were summarized, including IL1RL1, IL1R2, IL1R1, IL18R1, IL18RAP,SCL9A6, SCL9A4 and SH3YL1 (Supplementary Table S1).

**Supplemental Text S3. Soluble ST2 measurement**

For plasma samples, whole blood was drawn into sodium citrate tubes, processed immediately into plasma and blood cells, and stored at −80°C. Circulating sST2 was measured using a duoset ELISA kit (DY523B-05; R&D Systems, Minneapolis, MN, USA) according to the manufacturer’s instructions. Briefly, a 96-well microtiter plate was coated and incubated overnight at 4°C with 100 μL of capture antibody at a concentration of 1.0 μg/mL. Phosphate-buffered saline Tween-20 (PBST) was used as the washing buffer, comprising phosphate-buffered saline (PBS) containing 0.1% Tween-20. All washing steps were carried out three times between steps. Wells were blocked with 300 μL of 1% bovine serum albumin (BSA) in PBST for 2 h at 37°C. Either 100 μL of a diluted standard (ranging between 62.5 and 2000 pg/mL, seven dilutions) or 100 μL of a plasma sample (in 40-fold dilutions) was added and incubated for 2 h at room temperature. The plate was treated with a second biotinylated antibody (200 ng/mL) for 2 h and a solution of streptavidin-HRP was added, before 100 μL of substrate solution and 50 μL of stop solution were added. The absorbance at 450 nm was determined for each well using a spectra reader (Multiskan MK3 microplate reader; Thermo Scientific). The standard curve was fitted with the 4-parameter logistic method by Origin Lab 2016. The limit of detection for sST2 is 0.019 ng/mL, with mean intra-assay coefficient of variation of <6.0% and mean inter-assay coefficient of variation of <9.5%.

We have verified the difference in the detection of sST2 in different kits in our previous studies^1^. The Presage assay, currently recommended by the US Food and Drug Administration, was not used because it is not validated with our retrospective citrated plasma. We therefore evaluated DY523B-05 by R&D Systems based on the measurement range, recovery ration, limit of detection and required blood sample type before applied in our study. In addition, to verify and calibrate the different assay methods to measure sST2, we compared sST2 plasma concentrations measured with the R&D Systems assay vs. the Presage assay.

**Supplemental Text S4. Aortic dissection population**

Since Beijing Anzhen Hospital is the referral center for the disease of large vessel in China, the patients with aortic dissection come from all provinces of the country. Our patients were mainly from Hebei Province (n = 323,35.9%), Beijing City (n = 158,17.6%), Inner Mongolia Autonomous Region (n = 97,10.8%) (Supplemental Figure. S2). See Figure 1 for the total number of AD patients admitted to Anzhen hospital every year. In addition, according to the annual report of Anzhen Hospital, more than 3000 AD patients are treated and return visit in Anzhen Hospital every year. For aortic dissection, there is little epidemiological information about aortic dissection in the mainland of China. The estimated incidence of aortic dissection is 2.8/100000 (95% CI: 1.9-3.6) by sampling medical insurance data and pre hypothetical pre-treatment mortality ratio, which is higher in men than in women (3.7:1.5, P < 0.001). The average age was 58.9 ± 13.4 years ^2^.

**Supplemental Table S1.** Study characteristics.

| First author(year) | Country | Ethnicity | Age(mean±SD or range) | Case/control | Disease | Genes in the study |
| --- | --- | --- | --- | --- | --- | --- |
| Fangqin Wu  (2017)^3^ | China | Chinese | 58.2±11.7 | 0/50 | Hypertension | IL1RL1 |
| G. A. Queiroz  (2017)^4^ | Brazil | Brazilian | 4-11 | 0/1,223 | Asthma | IL1RL1 |
| Jengfeng Lin(2017)^5^ | China | Chinese | 43.5±17.8 | 618/601 | Coronary and peripheral artery disease | IL1RL1 |
| Zhongheng Wei(2017)^6^ | China | Chinese | 57.0±10.4 | 511/592 | Hepatocellular carcinoma | IL1RL1 |
| Hideki Inoue(2017)^7^ | JAPAN | Japanese | 55.0±16.2 | 110/126 | Asthma | IL1RL1 |
| Xin Long (2016)^8^ | China | Chinese | 50.1±13.1 | 646/301 | Schistosomiasis | IL1RL1 |
| Olga E. M. Savenije(2011)^9^ | Netherlands | Dutch | 0-8 | 0/4,146 | Asthma | IL1RL1 |
| Jennifer E. Ho(2013)^10^ | America | American | 58.6±10.0 | 0/2,991 | Control | IL1RL1; IL18R1; IL18RAP; IL1R1; IL1RL1; IL1RL2; SLC9A2; SLC9A4 |
| Russell S. Traister (2015)^11^ | America | American | 26.3±11.5 | 60/22 | Asthma | IL1RL1 |
| Makiko Shimizu(2005)^12^ | JAPAN | Japanese | 37.1±6.0 | 452/636 | Atopic dermatitis | -226999G/A* |
| David Díaz-Jiménez(2017)^13^ | Chile | Chilean | 41.6±3.8 | 192/137 | Ulcerative colitis | IL1RL1 |
| M. Ali (2008)^14^ | Australia | Caucasian | 58.2±11.7 | 0/50 | Hypertension | -226999G/A* |

*These two studies reported that –226999 G/A (rs6742278, in the SH3YL1 gene) is located in the distal promoter region of the ST2 gene

**Supplemental Table S2.** Hardy–Weinberg equilibrium.

| SNP | Gene | Genotype | Controls  (N=435) | | AD patients  (N=435) | |
| --- | --- | --- | --- | --- | --- | --- |
|  |  |  | Number | P value | Number | P value |
| rs4241211 | SLC9A4 | GG/GT/TT | 85/210/138 | 0.751 | 93/200/141 | 0.163 |
| rs3821204 | IL1RL1 | GG/GC/CC | 47/177/210 | 0.294 | 32/175/228 | 0.842 |
| rs3771172 | IL18R1 | TT/CT/CC | 42/180/210 | 0.706 | 31/167/236 | 0.845 |
| rs3917296 | IL1R1 | GG/AG/AA | 15/102/318 | 0.063 | 12/127/295 | 0.705 |
| rs11692304 | SLC9A4 | AA/GA/GG | 17/123/293 | 0.370 | 18/139/275 | 0.934 |
| rs2241132 | IL1RL2 | AA/CA/CC | 43/174/216 | 0.364 | 30/172/224 | 0.698 |
| rs2241116 | IL18R1 | AA/CA/CC | 15/125/294 | 0.704 | 11/129/295 | 0.482 |
| rs17775170 | SLC9A2 | AA/AG/GG | 18/121/294 | 0.222 | 14/123/297 | 0.772 |
| rs3917254 | IL1R1 | AA/AG/GG | 25/154/253 | 0.807 | 20/134/279 | 0.451 |
| rs6751967 | IL1RL1 | CC/TC/TT | 7/101/325 | 0.791 | 12/106/313 | 0.409 |
| rs887971 | IL18RAP | CC/CT/TT | 43/191/198 | 0.757 | 39/172/215 | 0.588 |
| rs4988958 | IL1RL1 | CC/TC/TT | 7/99/326 | 0.869 | 12/109/312 | 0.509 |
| rs4851608 | SLC9A4 | TT/TC/CC | 46/185/202 | 0.707 | 46/181/206 | 0.509 |
| crs1468788 | SLC9A4 | TT/TC/CC | 28/137/267 | 0.074 | 19/137/277 | 0.694 |
| rs1921622 | IL1RL1 | AA/GA/GG | 55/207/171 | 0.532 | 58/189/184 | 0.395 |
| rs13019803 | IL1R1 | TT/TC/CC | 9/135/291 | 0.141 | 6/92/337 | 0.922 |
| rs10167431 | IL1RL2 | TT/CT/CC | 53/187/193 | 0.461 | 56/191/184 | 0.562 |
| rs12712135 | IL1RL1 | GG/GA/AA | 81/198/116 | 0.835 | 112/195/125 | 0.045 |
| rs1558650 | IL18RAP | AA/AT/TT | 84/230/120 | 0.161 | 94/203/138 | 0.234 |
| rs6724322 | SLC9A4 | TT/CT/CC | 88/49/294 | <0.001 | 62/75/293 | <0.001 |
| rs11123923 | IL1RL1 | AA/CA/CC | 88/153/193 | <0.001 | 93/145/193 | <0.001 |

**Supplemental Table S3.** 19 tag SNPs associated with sST2 concentrations in controls in the discovery stage.

| SNP | Gene | Major allele | Minor allele | Beta^#^ | Se | P value* |
| --- | --- | --- | --- | --- | --- | --- |
| rs1921622 | IL1RL1 | G | A | -0.136 | 0.011 | 7.98E-30 |
| rs887971 | IL18RAP | T | C | -0.133 | 0.012 | 5.91E-27 |
| rs1558650 | IL18RAP | T | A | -0.129 | 0.011 | 1.21E-27 |
| rs3821204 | IL1RL1 | C | G | -0.120 | 0.011 | 3.98E-23 |
| rs3771172 | IL18R1 | C | T | -0.118 | 0.012 | 5.62E-21 |
| rs4851608 | SLC9A4 | C | T | -0.106 | 0.012 | 1.49E-17 |
| rs4241211 | SLC9A4 | T | G | -0.099 | 0.011 | 3.05E-17 |
| rs2241116 | IL18R1 | C | A | -0.105 | 0.015 | 8.20E-12 |
| rs1468788 | SLC9A4 | C | T | -0.109 | 0.013 | 1.38E-15 |
| rs12712135 | IL1RL1 | A | G | -0.054 | 0.013 | 1.90E-05 |
| rs11692304 | SLC9A4 | G | A | -0.081 | 0.015 | 1.08E-07 |
| rs2241132 | IL1RL2 | C | A | -0.044 | 0.013 | 6.50E-04 |
| rs17775170 | SLC9A2 | G | A | -0.059 | 0.015 | 1.07E-04 |
| rs13019803 | IL1R1 | C | T | -0.069 | 0.016 | 2.90E-05 |
| rs6751967 | IL1RL1 | T | C | -0.055 | 0.018 | 2.41E-03 |
| rs4988958 | IL1RL1 | T | C | -0.054 | 0.018 | 3.06E-03 |
| rs3917254 | IL1R1 | G | A | -0.038 | 0.014 | 7.71E-03 |
| rs10167431 | IL1RL2 | T | C | 0.037 | 0.013 | 3.20E-03 |
| rs3917296 | IL1R1 | A | G | -0.021 | 0.016 | 1.90E-01 |

MAF: minor allele frequency

# beta estimate represents estimated change in log-sST2 per minor allele.

* Significant after Bonferroni correction for 19 tests (*P*< 0.0026)

**Supplemental Table S4.** Summary of type A aortic dissection characteristics.

|  | Overall† | | | |
| --- | --- | --- | --- | --- |
|  | sST2 ＜52.8 ng/ml^&^ | sST2≥52.8ng/ml^&^ | P value |  |
|  | (N=231) | (N=232) |  |  |
| log-sST2 | 1.34(0.27) | 2.12 (0.31) | <0.001 |  |
| Age | 49.20 (11.85) | 50.36(10.61) | 0.267 |  |
| Sex (male) | 165 (71.4%) | 180 (77.6%) | 0.128 |  |
| Smoke (current) | 98 (42.4%) | 116 (50.0%) | 0.102 |  |
| CAD (yes) | 24 (10.4%) | 23(9.9%) | 0.865 |  |
| Diabetes (yes) | 14 (6.1%) | 10 (4.3%) | 0.396 |  |
| Hypertension (yes) | 162 (70.1%) | 174 (75.0%) | 0.240 |  |
| Hyperlipidemia (yes) | 53 (22.9%) | 44 (19.0%) | 0.293 |  |
| BMI (kg/m^2^) | 26.15 (3.91) | 26.28(3.24) | 0.696 |  |
| Marfan (yes) | 5 (2.2%) | 1 (0.4%) | 0.122 |  |
| BAV (yes) | 4 (1.7%) | 2 (0.9%) | 0.450 |  |
| Heart function (disorder) | 75(32.5%) | 75 (32.3%) | 0.974 |  |
| Aortic aneurysm | 95 (41.1%) | 93 (40.1%) | 0.820 |  |
| Acuity |  |  | <0.001 |  |
| Acute | 185 (80.1%) | 228 (98.3%) |  |  |
| Subacute | 32 (13.9%) | 0 (0.0%) |  |  |
| Chronic | 14 (6.1%) | 4 (1.7%) |  |  |
| Chest/back pain (acute) | 139 (60.2%) | 162 (69.8%) | 0.029 |  |
| Severely involved aortic root | 80 (34.6%) | 87 (37.5%) | 0.521 |  |
| Severely involved distal aorta | 182 (78.8%) | 205 (88.4%) | 0.005 |  |
| Malperfusion before surgery(yes) | 54 (23.4%) | 113 (48.7%) | <0.001 |  |
| Malperfusion before surgery |  |  |  |  |
| Coronary | 5 (2.2%) | 37 (15.9%) | <0.001 |  |
| Mesentery | 18 (7.8%) | 20 (8.6%) | 0.745 |  |
| Nervous system | 25 (10.8%) | 28 (12.1%) | 0.674 |  |
| Renal | 18 (7.8%) | 64 (27.6%) | <0.001 |  |
| Hepatic | 2 (0.9%) | 29 (12.5%) | <0.001 |  |

† The sST2 concentration was measured in 463 type A AD patients in the two stage.

**Supplemental Table S5.** rs13019803 associated with sST2 concentrations in AD patients in the discovery stage after adjusting confounders.

|  | Beta( 95%CI)^a^ | P value^a^ |
| --- | --- | --- |
| Overall (N=342) | 0.120(0.023,0.216) | 0.015 |
| Type A (N=173) | 0.198(0.042,0.353) | 0.013 |
| Type B (N=169) | -0.020(-0.127,0.087) | 0.712 |

^a^Adjusted age, sex, BMI, hypertension, hyperlipidemia, diabetes, smoking, cardiovascular disease, acute, abrupt onset pain , severely involved distal aorta and pre-operative presence of malperfusion.

.**Supplemental Table S6.** Baseline characters of rs13019803 genotypes in type A AD patients (N=652).

|  | CC(N=498) | CT (N=142) | TT (N=12) | P value |
| --- | --- | --- | --- | --- |
| Age | 49.79(11.93) | 48.29(10.98) | 55.92(10.09) | 0.068 |
| Sex, male | 374(75.1%) | 115(81.0%) | 7(58.3%) | 0.121 |
| BMI(kg/m^2^) | 26.10(3.64) | 25.79(3.61) | 26.38(4.11) | 0.623 |
| Smoke(yes) | 201(40.4%) | 73(51.4%) | 5(41.7%) | 0.063 |
| Diabetes(yes) | 29(5.8%) | 6(4.2%) | 1(8.3%) | 0.696 |
| CAD(yes) | 42(8.4%) | 10(7.0%) | 1(8.3%) | 0.866 |
| Hyperlipidemia(yes) | 88(17.7%) | 26(18.3%) | 1(8.3%) | 0.684 |
| Hypertension (yes) | 353(70.9%) | 99(69.7%) | 11(91.7%) | 0.272 |
| Log-sST2† | 1.77(0.49) | 1.58(0.44) | 1.84(0.42) | 0.002 |

AD: aortic dissection; BMI: body mass index; CAD: coronary artery disease.

† The sST2 concentration was measured in 463 type A AD patients in the two stage, including TT genotype =5, CT=101, CC=357.

**Supplemental Table S7.** Subgroup analysis of the association between rs13019803C and the risk of type A AD patients

|  | OR( 95%CI))* | P value |
| --- | --- | --- |
| Hypertension^&^ |  |  |
| Model 1 | 1.68(1.26,2.25) | <0.001 |
| Model 2 | 1.69(1.27,2.26) | <0.001 |
| Model 3 | 1.71(1.26,2.33) | 0.001 |
| Non-hypertension^&^ |  |  |
| Model 1 | 1.61(1.16,2.25) | 0.005 |
| Model 2 | 1.56(1.11,2.18) | 0.010 |
| Model 3 | 1.59(1.13,2.23) | 0.007 |

* C allele is risk allele.

& There were 268 controls and 463 AAD patients with hypertension; and 1110 controls and 189 AAD patients without hypertension.

Model 1: Unadjusted.

Model 2: Adjusted age, sex.

Model 3: Adjusted age, sex, BMI, hyperlipidemia, diabetes, smoking, and cardiovascular disease.

**Supplemental Figure S1.** Systematic review of the literature about the association between genetic variance and sST2 concentrations.


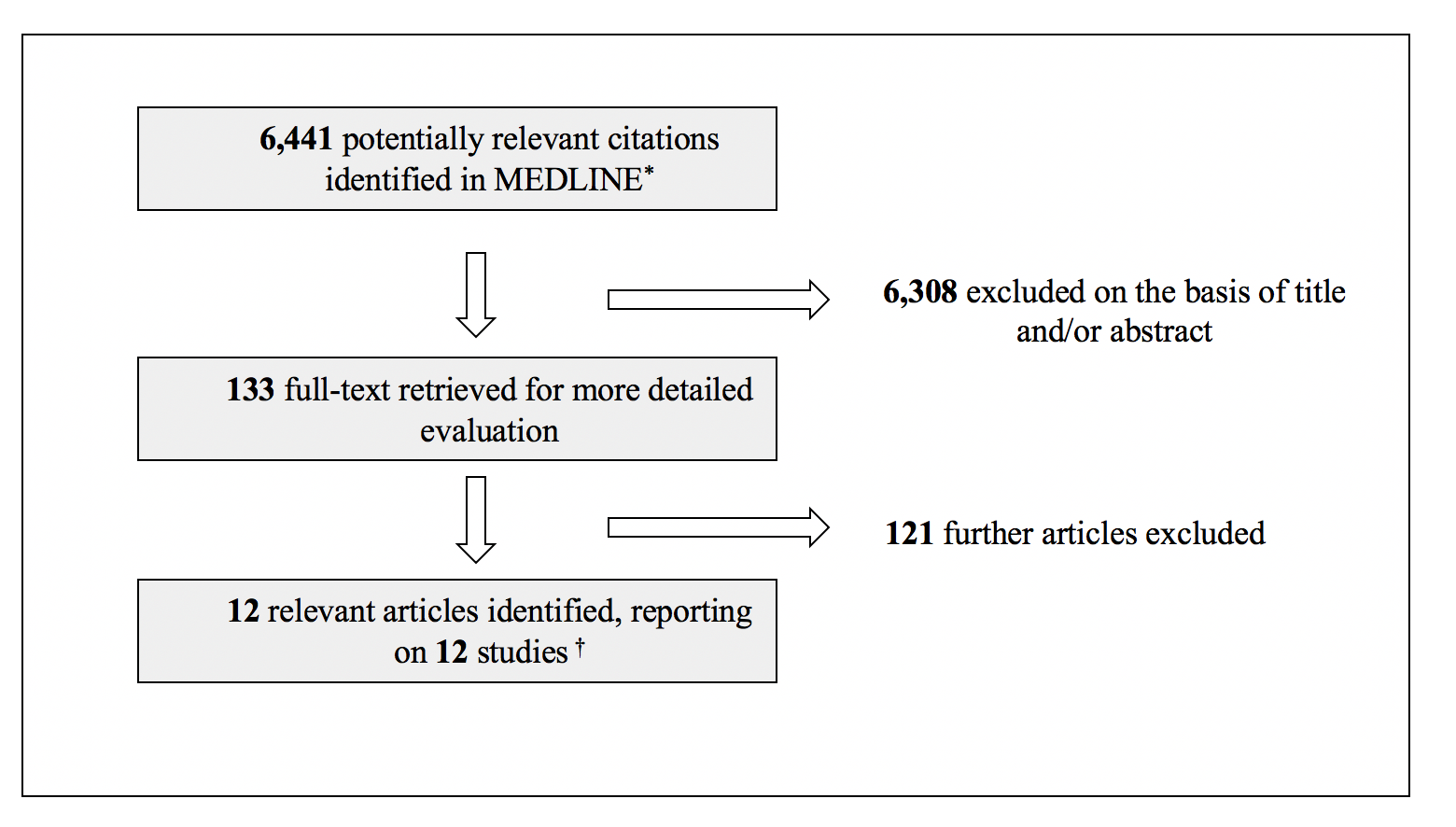


* The search strategy is shown in Supplemental Text S1.

† Details of the 12 studies are shown in Supplemental Table S1.

**Supplemental Figure S2.** Study design and population.

**
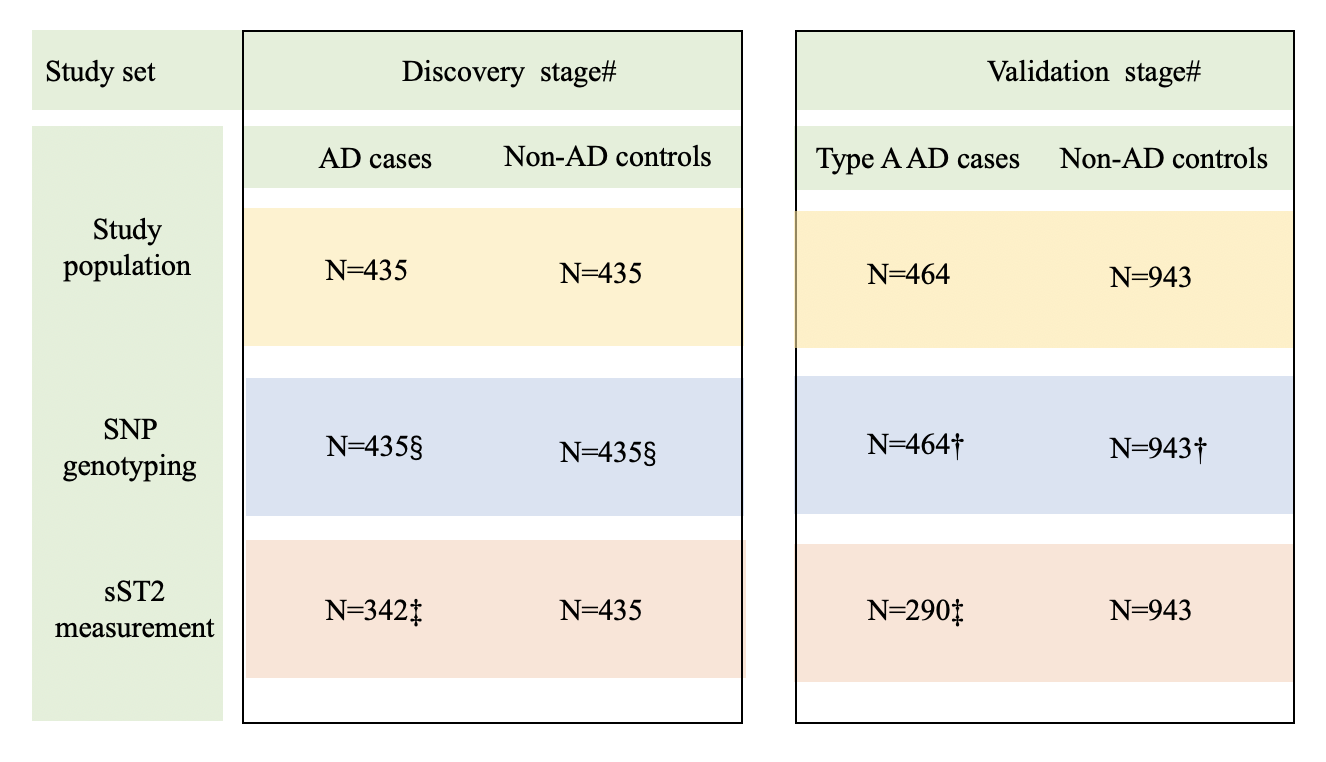
**

# Details of discovery and validation stages are shown in Fig. 1; § 21 SNPs were genotyped; † One SNP (rs13019803) was genotyped; ‡ The sST2 concentration was measured in 342 AD patients in the discovery stage and in 290 Type A AD patients in the validation stage with plasma samples were available.

**Supplemental Figure S3.** Province of aortic dissection patient

**
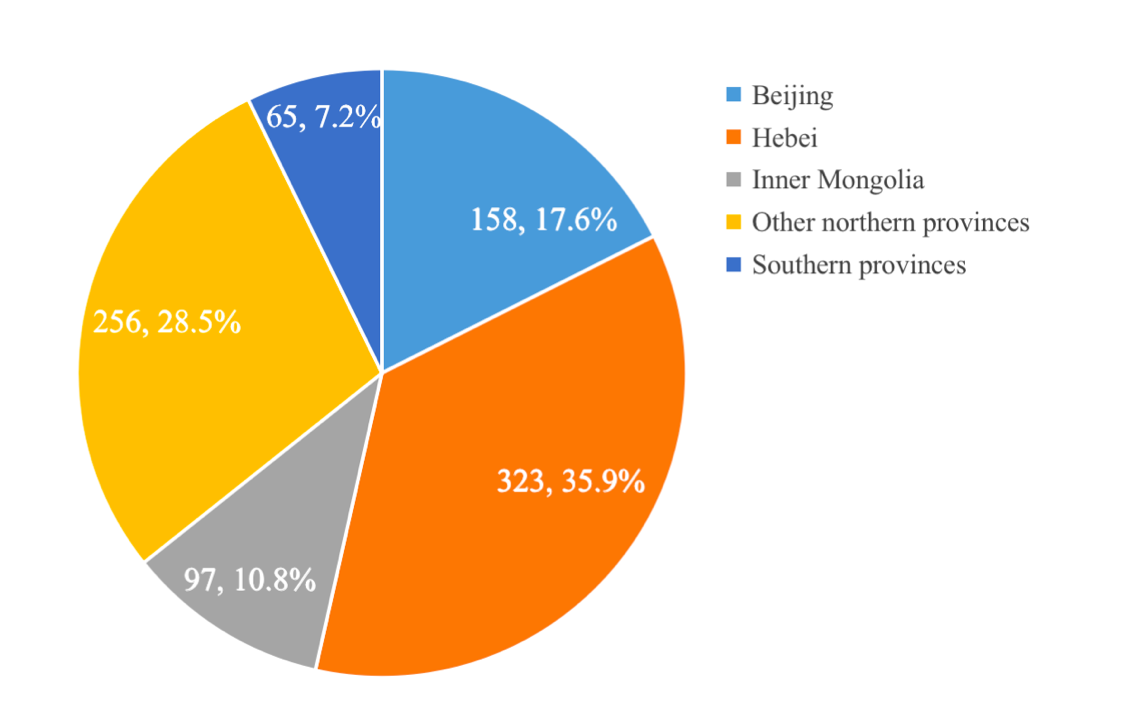
**

**Reference:**

1. Wang Y, Tan X, Gao H, Yuan H, Hu R, Jia L, et al. Magnitude of Soluble ST2 as a Novel Biomarker for Acute Aortic Dissection. (2018).Circulation.137(3):259-69. doi:10.1161/CIRCULATIONAHA.117.030469

2. Xia L, Li JH, Zhao K, Wu HY. Incidence and in-hospital mortality of acute aortic dissection in China: analysis of China Health Insurance Research (CHIRA) Data 2011. (2015).J Geriatr Cardiol.12(5):502-6. doi:10.11909/j.issn.1671-5411.2015.05.021

3. Wu F, Li L, Wen Q, Yang J, Chen Z, Wu P, et al. A functional variant in ST2 gene is associated with risk of hypertension via interfering MiR-202-3p. (2017).Journal of cellular and molecular medicine.21(7):1292-9. doi:10.1111/jcmm.13058

4. Queiroz GA, Costa RS, Alcantara-Neves NM, Nunes de Oliveira Costa G, Barreto ML, Carneiro VL, et al. IL33 and IL1RL1 variants are associated with asthma and atopy in a Brazilian population. (2017).Int J Immunogenet.44(2):51-61. doi:10.1111/iji.12306

5. Lin JF, Wu S, Juang JJ, Chiang FT, Hsu LA, Teng MS, et al. IL1RL1 single nucleotide polymorphism predicts sST2 level and mortality in coronary and peripheral artery disease. (2017).Atherosclerosis.257:71-7. doi:10.1016/j.atherosclerosis.2016.12.020

6. Wei ZH, Li YY, Huang SQ, Tan ZQ. Genetic variants in IL-33/ST2 pathway with the susceptibility to hepatocellular carcinoma in a Chinese population. (2018).Cytokine. doi:10.1016/j.cyto.2018.03.036

7. Inoue H, Ito I, Niimi A, Matsumoto H, Oguma T, Tajiri T, et al. Association of interleukin 1 receptor-like 1 gene polymorphisms with eosinophilic phenotype in Japanese adults with asthma. (2017).Respir Investig.55(6):338-47. doi:10.1016/j.resinv.2017.08.006

8. Long X, Daya M, Zhao J, Rafaels N, Liang H, Potee J, et al. The role of ST2 and ST2 genetic variants in schistosomiasis. (2017).J Allergy Clin Immunol.140(5):1416-22 e6. doi:10.1016/j.jaci.2016.12.969

9. Savenije OE, Kerkhof M, Reijmerink NE, Brunekreef B, de Jongste JC, Smit HA, et al. Interleukin-1 receptor-like 1 polymorphisms are associated with serum IL1RL1-a, eosinophils, and asthma in childhood. (2011).J Allergy Clin Immunol.127(3):750-6 e1-5. doi:10.1016/j.jaci.2010.12.014

10. Ho JE, Chen WY, Chen MH, Larson MG, McCabe EL, Cheng S, et al. Common genetic variation at the IL1RL1 locus regulates IL-33/ST2 signaling. (2013).J Clin Invest.123(10):4208-18. doi:10.1172/JCI67119

11. Traister RS, Uvalle CE, Hawkins GA, Meyers DA, Bleecker ER, Wenzel SE. Phenotypic and genotypic association of epithelial IL1RL1 to human TH2-like asthma. (2015).J Allergy Clin Immunol.135(1):92-9. doi:10.1016/j.jaci.2014.06.023

12. Shimizu M, Matsuda A, Yanagisawa K, Hirota T, Akahoshi M, Inomata N, et al. Functional SNPs in the distal promoter of the ST2 gene are associated with atopic dermatitis. (2005).Human molecular genetics.14(19):2919-27. doi:10.1093/hmg/ddi323

13. Diaz-Jimenez D, Nunez L, De la Fuente M, Dubois-Camacho K, Sepulveda H, Montecino M, et al. A functional IL1RL1 variant regulates corticosteroid-induced sST2 expression in ulcerative colitis. (2017).Scientific reports.7(1):10180. doi:10.1038/s41598-017-10465-0

14. Ali M, Zhang G, Thomas WR, McLean CJ, Bizzintino JA, Laing IA, et al. Investigations into the role of ST2 in acute asthma in children. (2009).Tissue antigens.73(3):206-12. doi:10.1111/j.1399-0039.2008.01185.x
